# Bioprospecting of Marine Organisms: Exploring Antibacterial Activities in Aqueous and Organic Extracts

**DOI:** 10.3390/microorganisms13040940

**Published:** 2025-04-18

**Authors:** Vinícius Paulino Pinto Menezes, Aldeni Moreira da Silva Filho, Aline Jeferson Costa, Elielton Nascimento, Ulisses Santos Pinheiro, Renata Pinheiro Chaves, Alexandre Lopes Andrade, Mayron Alves de Vasconcelos, Edson Holanda Teixeira, Alexandre Holanda Sampaio, Celso Shiniti Nagano, Rômulo Farias Carneiro

**Affiliations:** 1 Laboratório de Biotecnologia Marinha—BioMar, Departamento de Engenharia de Pesca, Universidade Federal do Ceará, Av. Humberto Monte, s/n, Campus do Pici, bloco 871, Fortaleza 60440-970, CE, Brazil; vinicius.paulino@alu.ufc.br (V.P.P.M.); aldenibastos4@gmail.com (A.M.d.S.F.); alinejcosta@alu.ufc.br (A.J.C.); renatapinheirochaves@gmail.com (R.P.C.); alexholandasampaio@gmail.com (A.H.S.);; 2 Departamento de Zoologia, Universidade Federal de Pernambuco, Av. Prof. Moraes Rego, 1235, Cidade Universitária, Recife 50670-901, PE, Brazil; biologoefn@gmail.com (E.N.); uspinheiro@hotmail.com (U.S.P.); 3 Laboratório Integrado de Biomoléculas—LIBS, Departamento de Patologia e Medicina Legal, Universidade Federal do Ceará, Av. Monsenhor Furtado, s/n, Fortaleza 60430-160, CE, Brazil; alexandre.andrade@uece.br (A.L.A.); mayronvasconcelos@gmail.com (M.A.d.V.); edsonlec@gmail.com (E.H.T.); 4 Faculdade de Educação de Itapipoca, Universidade Estadual do Ceará, Av. da Universidade, s/n, Madalenas, Itapipoca 62500-000, CE, Brazil

**Keywords:** antibacterial activity, bioactive extracts, marine organisms

## Abstract

This study evaluated the antibacterial activity of aqueous and organic extracts from 78 marine organisms, including seaweeds and sponges, collected along the coast of Ceará, Brazil. Extracts were obtained by maceration using distilled water and 50% acetonitrile and tested against *Staphylococcus aureus*, *Staphylococcus epidermidis* (Gram-positive), and *Escherichia coli* (Gram-negative) using the disk diffusion method, and inhibition zone diameters were measured. Antibacterial activity was observed in 30.7% of the extracts, with organic extracts showing higher efficacy. Several sponge species, particularly those from the genus *Aplysina*, as well as *Amphimedon compressa*, *Amphimedon viridis*, *Mycale* sp., and *Pseudosuberites* sp., demonstrated notable inhibitory effects. While seaweed aqueous extracts showed no activity, some organic extracts—especially from *Amansia multifida*—were effective against Gram-positive strains. In general, Gram-positive bacteria were more susceptible than Gram-negative bacteria. These findings underscore the biotechnological potential of marine organisms from the Brazilian coast as promising sources of antibacterial compounds and support future efforts focused on the isolation, characterization, and toxicological evaluation of active metabolites for pharmaceutical and industrial applications.

## 1. Introduction

Marine organisms are a prolific source of natural products, producing structurally diverse bioactive compounds shaped by environmental conditions distinct from those in terrestrial ecosystems. These compounds, typically derived from secondary metabolism, are not directly involved in growth or maintenance but play key roles in mediating ecological interactions. Many of these molecules exhibit significant biological activities and have demonstrated therapeutic potential against various pathogens [1,2,3].

Marine-derived compounds such as peptides, alkaloids, polyketides, and terpenes have attracted considerable attention due to their broad spectrum of bioactivity and chemical diversity. In particular, antibacterial compounds from marine sources are of growing interest as potential tools to combat antibiotic resistance [4,5]. Currently, 33 marine bioactive compounds are at different stages of drug development—including preclinical and clinical phases I–III—and 15 have been approved by regulatory agencies in the United States, Australia, Japan, and China. Notably, the nucleoside Ara-A, isolated from a marine sponge, has been approved for antiviral treatment [6,7]. Furthermore, 11 compounds in the preclinical pipeline—primarily from the shikimate, peptide, polyketide, alkaloid, and terpene classes—have shown antibacterial activity and are derived from marine sponges, algae, bacteria, bryozoans, and soft corals [6,7].

The growing inefficacy of conventional antibiotics due to bacterial resistance poses a major global health threat. Resistance may arise through the acquisition of resistance genes or the occurrence of spontaneous mutations. The inappropriate or excessive use of antibiotics accelerates this process, leading to treatment failures and an increased burden on healthcare systems, along with higher mortality rates [8]. In this context, the discovery of novel antibacterial molecules is critical to developing alternative therapeutic strategies.

Recent studies have explored the antibacterial properties of extracts from a wide range of marine organisms, including invertebrates and macroalgae, targeting both common and multidrug-resistant bacterial strains [9,10,11,12,13]. These investigations underscore the importance of marine bioprospecting for the identification of secondary metabolites with biotechnological potential. Among screening methodologies, the disk diffusion assay remains a widely employed approach for the preliminary evaluation of antibacterial activity, owing to its simplicity, low cost, and ability to generate rapid and semi-quantitative results. When using this method, sterile paper disks impregnated with test extracts are placed on agar plates inoculated with bacterial cultures. After incubation, the zones of inhibition surrounding the disks are measured to assess antimicrobial potential against specific bacterial strains [14].

In this study, we investigated the antibacterial activity of aqueous and organic extracts from marine organisms collected along the coast of Ceará, Northeastern Brazil. As part of a broad bioprospecting effort, we aimed to identify extracts capable of inhibiting both Gram-positive and Gram-negative bacteria. The results are expected to support future efforts directed at achieving the isolation, structural characterization, and development of novel bioactive compounds with potential applications in biotechnology and medicine.

## 2. Materials and Methods

### 2.1. Material Collection

Marine macroalgae and sponges were manually collected from the beaches of Pacheco and Paracuru and by scuba diving at Parque da Pedra da Risca do Meio, along the coast of Ceará, Brazil (Tables S1–S3). Fragments of the collected organisms were individually placed in plastic tubes and kept cool in thermal boxes during transport to the Marine Biotechnology Laboratory (BioMar-Lab), Department of Fisheries Engineering, Federal University of Ceará. All collections and the use of biological material were authorized by the Brazilian environmental regulatory systems SISBIO (Biodiversity Authorization and Information System, ID: 33913-10, 33913-11) and SISGEN (National System for Genetic Heritage and Associated Traditional Knowledge Management, ID: AC14AF9, A9D15EA, A1792FE, AC71058, A625FEE, ACC97AD). Macrolgae were identified at the Department of Fisheries Engineering, Federal University of Ceará, and sponges were identified at the Department of Zoology, Federal University of Pernambuco.

Macroalgae were identified at the Department of Fisheries Engineering, Federal University of Ceará, and sponges were identified at the Department of Zoology, Federal University of Pernambuco.

### 2.2. Aqueous Extraction

Different tissues from the marine organisms were macerated with or without liquid nitrogen, depending on tissue consistency, and then homogenized in distilled water at a ratio of 1:3 (*w*/*v*) for macroalgae and 1:2 for sponges. Homogenization was performed using a refrigerated shaker (TH 6430B, Thoth Equipments, Piracicaba, Brazil) at 170 rpm for 4 h at 25 °C. The resulting extracts were centrifuged at 9000× *g* for 15 min, and the supernatants were transferred to new tubes and stored at −20 °C until further use.

### 2.3. Organic Extraction

Tissues were processed as described for aqueous extraction. The macerated material was homogenized in 50% acetonitrile at the same ratios (1:3 for macroalgae and 1:2 for sponges) and agitated at 170 rpm for 4 h at 25 °C. The aliquots (2 mL) of the resulting extracts were transferred to microtubes and concentrated by acetonitrile evaporation using a vacuum concentrator (Labconco, Kansas City, MO, USA) at 35 °C for 2 h. The concentrated organic extracts were stored at −20 °C until further analysis.

### 2.4. Bacteria and Culture Conditions

To assess antibacterial activity, *Escherichia coli* ATCC 11303, *Staphylococcus aureus* ATCC 25923, and *Staphylococcus epidermidis* ATCC 12228 were obtained from the microbial collection of the Integrated Biomolecules Laboratory (LIBS), Department of Fisheries Engineering, Federal University of Ceará. Bacterial colonies were cultured on Petri dishes containing Tryptic Soy Agar (TSA; Sigma-Aldrich, St. Louis, MO, USA) and then inoculated into tubes with 15 mL of Tryptic Soy Broth (TSB; Sigma-Aldrich, MO, USA). Tubes were incubated at 37 °C for 18 h using an incubator (KM-CC17RU1A, Panasonic, Newark, NJ, USA) to promote bacterial growth.

#### 2.4.1. Antibiogram Assay

The bacterial concentration was adjusted to 2 × 10^8^ colony-forming units (CFUs)·mL^−^^1^ using a spectrophotometer at 620 nm based on calibration curves previously established for each strain. Antibacterial activity was assessed using a modified disk diffusion method based on Nugroho et al.’s protocol [12], with modifications. All samples and controls were exposed to ultraviolet light for 15 min to ensure sterility. Sterile 6 mm paper disks (Laborclin, Pinhais, Brazil) were placed on TSA plates previously seeded with bacterial suspensions, and 10 µL of each extract was applied to the disks. Negative controls included 10 µL of distilled water and 50% acetonitrile, while ampicillin (50 µg) served as a positive control. Plates were incubated at 37 °C for 20 h, after which inhibition zones were measured. The presence of inhibition zones indicated antibacterial activity, while their absence suggested bacterial resistance and a lack of activity in the tested extract.

#### 2.4.2. Statistical Analysis

All experiments were conducted in triplicate in three independent runs. Data were analyzed using one-way analysis of variance (ANOVA) followed by Tukey’s post hoc test using GraphPad Prism software (version 9.0; San Diego, CA, USA). Statistical significance was considered at *p* < 0.05.

## 3. Results

A total of 78 marine organisms were collected, of which the majority (65.4%) were marine algae. Among these, red algae were the most prevalent, representing 56.9% of all algae and 37.2% of the total specimens. Marine sponges comprised the remaining 34.6% of the samples. Disk diffusion assays were performed on both aqueous and organic extracts, with 10 aqueous extracts (12.8%) and 23 organic extracts (29.5%) exhibiting antibacterial activity against at least one tested bacterial strain (Tables S4 and S5).

Among the aqueous extracts, 37% of sponge-derived samples showed antibacterial activity, while none of the algal aqueous extracts were active. In contrast, organic extracts displayed higher antibacterial potential, with 48.1% of sponge and 19.6% of algal extracts showing inhibitory effects. Activity against the Gram-negative bacterium *Escherichia coli* was observed in 10.2% of the tested species. In comparison, Gram-positive strains were more frequently inhibited, with 24.4% of extracts effective against *Staphylococcus aureus* and 23.1% against *Staphylococcus epidermidis*.

**Figure 1:**
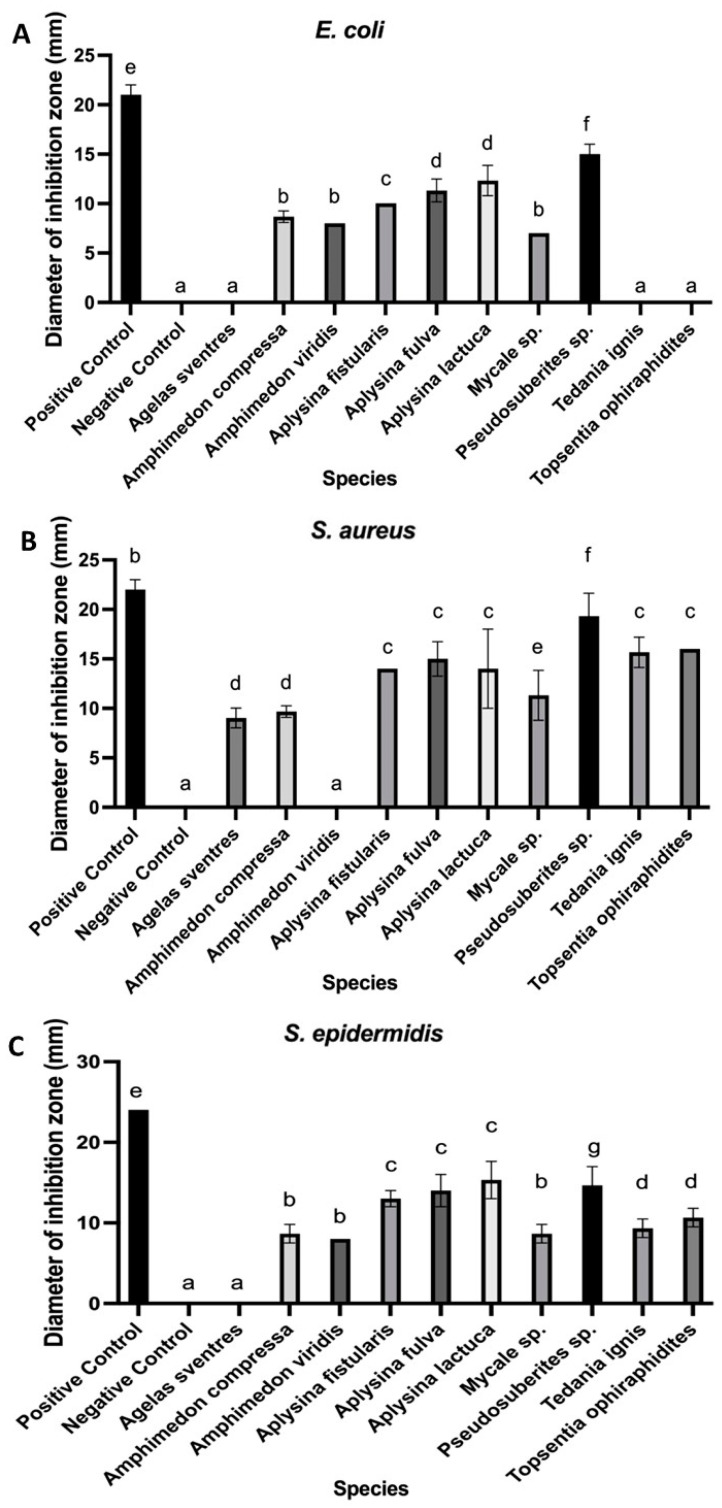
Antibacterial activity of aqueous extracts from marine organisms. Mean inhibition zone diameters (mm) include the disk diameter (6 mm). Disks were loaded with 10 µL of aqueous extract, distilled water (negative control), or ampicillin (50 µg; positive control). Panels show results against (**A**) *Escherichia coli*, (**B**) *Staphylococcus aureus*, and (**C**) *Staphylococcus epidermidis*. Different letters within each panel indicate statistically significant differences (*p* < 0.05).

**Figure 2:**
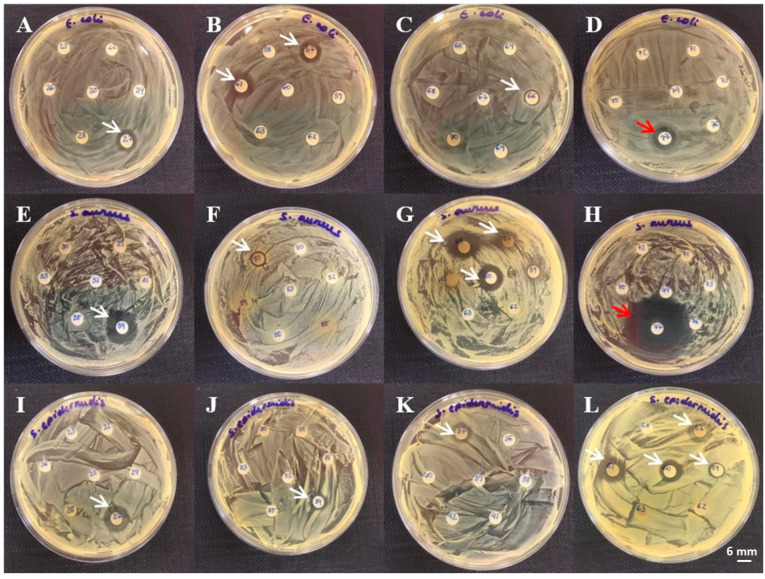
Representative results of disk diffusion assays for aqueous extracts of marine organisms against *E. coli*, *S. aureus*, and *S. epidermidis*. Panels (**A**–**D**): *E. coli*; (**E**–**H**): *S. aureus*; (**I**–**L**): *S. epidermidis*. Red arrows indicate inhibition zones produced by ampicillin (50 µg); white arrows indicate inhibition zones produced by the extracts. (**A**): *Aplysina fistularis*; (**B**): *Aplysina fulva*, *Pseudosuberites* sp.; (**C**): *Amphimedon compressa*; (**D**): ampicillin (50 µg); (**E**): *Tedania ignis*; (**F**): *Ircinia felix*; (**G**): *Aplysina fulva*, *Mycale* sp., *Topsentia ophiraphidites*; (**H**): ampicillin (50 µg); (**I**): *Aplysina fistularis*; (**J**): *Tedania ignis*; (**K**): *Aplysina lactuca*; (**L**): *Aplysina fulva*, *Mycale* sp., *Topsentia ophiraphidites*, *Pseudosuberites* sp.

Among the aqueous extracts, the most pronounced inhibition zones were observed in the extract from *Pseudosuberites* sp., with diameters of 15.00 ± 1.00 mm (*E. coli*), 19.33 ± 2.31 mm (*S. aureus*), and 14.67 ± 2.31 mm (*S. epidermidis*) (Figure 1). Six sponge extracts—*Amphimedon compressa*, *Aplysina fistularis*, *Aplysina fulva*, *Aplysina lactuca*, *Mycale* sp., and *Pseudosuberites* sp.—were active against all three bacterial strains, representing 60% of the active aqueous samples. In contrast, *Agelas sventres* displayed the lowest activity, inhibiting only *S. aureus* (9.00 ± 1.00 mm). *Tedania ignis* and *Topsentia ophiraphidites* showed no activity against *E. coli*. Examples of inhibition halos from aqueous extracts are shown in Figure 2.

**Figure 3:**
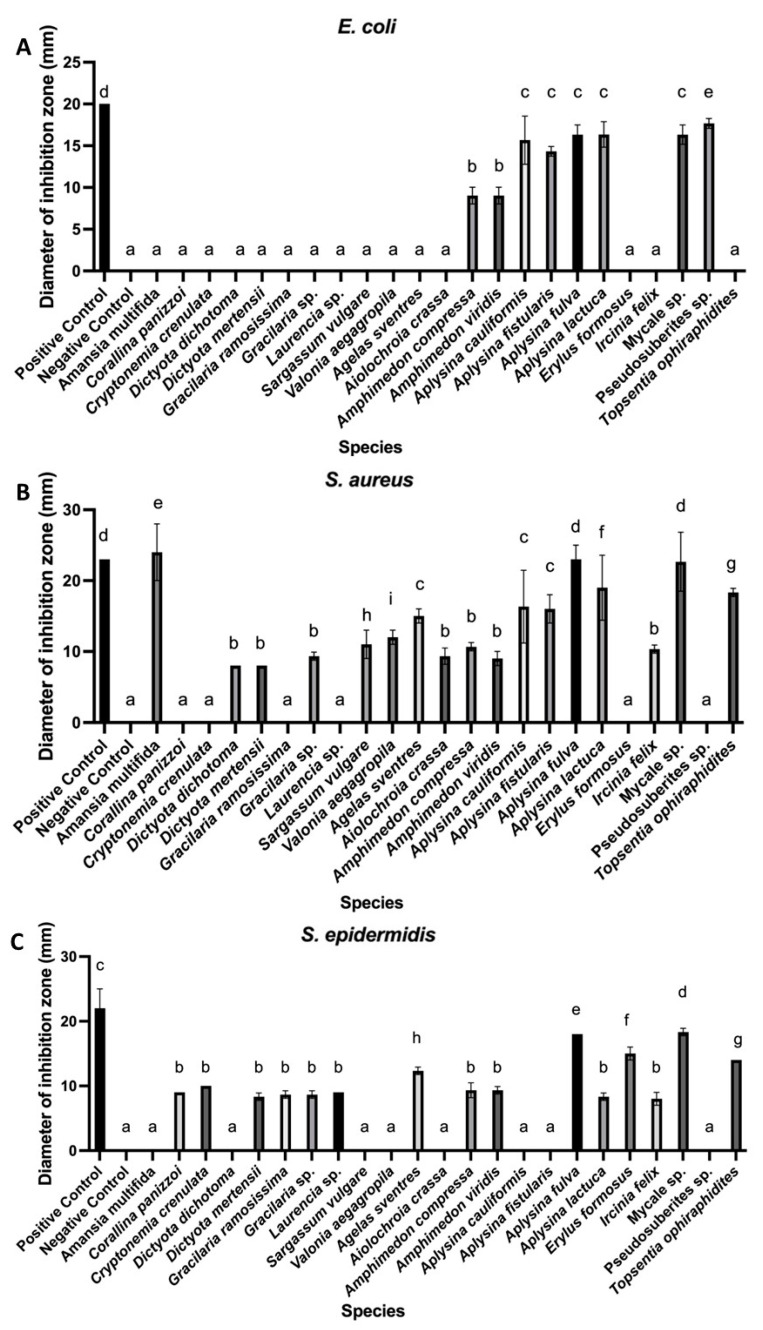
Antibacterial activity of organic extracts from marine organisms. Mean inhibition zone diameters (mm) include the disk diameter (6 mm). Disks were loaded with 10 µL of organic extract, distilled water (negative control), or ampicillin (50 µg; positive control). Panels show results against (**A**) *E. coli*, (**B**) *S. aureus*, and (**C**) *S. epidermidis*. Different letters within each panel indicate statistically significant differences (*p* < 0.05).

The genus *Aplysina* showed the highest number of positive results in both extraction types, with inhibition zones ranging from 8.33 ± 0.58 to 23.00 ± 2.00 mm and consistent activity against all of the tested bacterial strains (Figure 1 and Figure 3). Other sponges, such as *Mycale* sp. and *Topsentia ophiraphidites*, also exhibited notable activity, particularly in organic extracts. Four sponge species—*Aiolochroia crassa*, *Aplysina cauliformes*, *Erylus formosus*, and *Ircinia felix*—that were inactive in aqueous extractions showed activity in their organic counterparts. Conversely, only *Tedania ignis* lost activity upon organic extraction, while the other nine species maintained or increased their antibacterial performance.

None of the aqueous algal extracts exhibited antibacterial activity. However, 19.6% of organic algal extracts were effective, primarily against *Staphylococcus* strains, with inhibition zones ranging from 8.00 ± 0.00 to 12.00 ± 1.00 mm (Figure 3). A notable exception was *Amansia multifida*, which produced a prominent inhibition zone of 24.00 ± 4.00 mm against *S. aureus*. Only two algal species—*Dictyota mertensii* and *Gracilariopsis* sp.—exhibited activity against both *Staphylococcus* strains, with inhibition zones ranging from 8.00 ± 0.00 to 9.33 ± 0.58 mm.

Organic extracts from *Amansia multifida* (24.00 ± 4.00 mm), *Dictyota dichotoma* (8.00 ± 0.00 mm), *Sargassum vulgare* (11.00 ± 2.00 mm), and *Valonia aegagropila* (12.00 ± 1.00 mm) were active exclusively against *S. aureus*. In contrast, extracts from *Corallina panizzoi* (9.00 ± 0.00 mm), *Cryptonemia crenulata* (10.00 ± 0.00 mm), *Gracilaria ramosissima* (8.67 ± 0.58 mm), and *Laurencia* sp. (9.00 ± 0.00 mm) were active only against *S. epidermidis*. Among the macroalgae, 42.8% of brown algae exhibited antibacterial activity, followed by 20.7% of red algae and only 6% of green algae.

**Figure 4:**
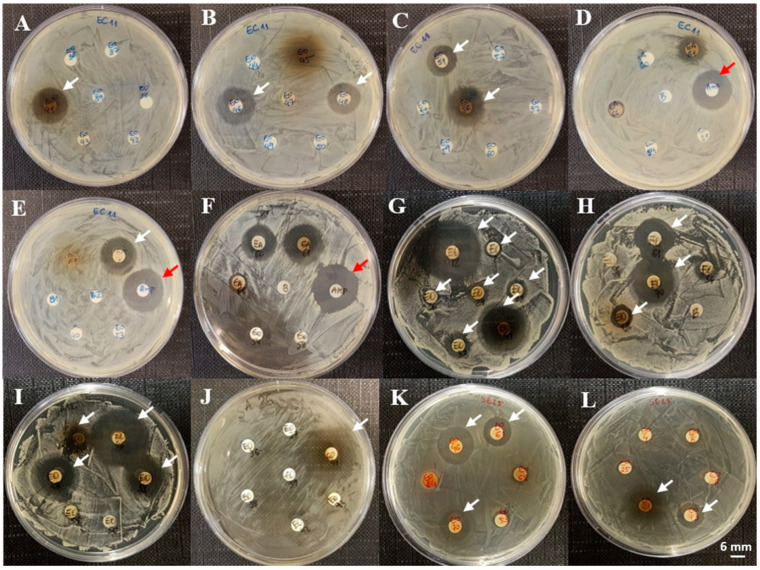
Representative results of disk diffusion assays for organic extracts of marine organisms against *E. coli*, *S. aureus*, and *S. epidermidis*. Panels (**A**–**E**): *E. coli*; (**F**–**J**): *S. aureus*; (**K**,**L**): *S. epidermidis*. Red arrows indicate inhibition zones from ampicillin (50 µg); white arrows indicate inhibition zones from the extracts. (**A**): *Aplysina fulva*; (**B**): *Mycale* sp., *Aplysina fistularis*; (**C**): *Aplysina cauliformes*, *Pseudosuberites* sp.; (**D**): ampicillin (50 µg); (**E**): *Aplysina lactuca*; (**F**): ampicillin (50 µg); (**G**): *Amansia multifida*, *Dictyota mertensii*, *D. dichotoma*, *Sargassum vulgare*, *Gracilaria ramosissima*, *Valonia aegagropila*, *Aplysina fulva*; (**H**): *Topsentia ophiraphidites*, *Ircinia felix*, *Aplysina lactuca*; (**I**): *Aiolochroia crassa*, *Mycale* sp., *Aplysina fistularis*, *A. cauliformes*; (**J**): *Aplysina fulva*; (**K**): *Mycale* sp., *Topsentia ophiraphidites*, *Aplysina lactuca*; (**L**): *Aplysina fulva*, *Erylus formosus*.

Among the sponge-derived organic extracts, the strongest inhibition zones were observed in *Aplysina fulva* (23.00 ± 2.00 mm) and *Mycale* sp. (22.67 ± 4.16 mm) against *S. aureus*. Activity against *E. coli* was observed in extracts from *Amphimedon compressa* and *Amphimedon viridis*, species of the genus *Aplysina*, *Mycale* sp., and *Pseudosuberites* sp., with inhibition zones ranging from 9.00 ± 1.00 to 17.67 ± 0.58 mm. Five sponge extracts (*Amphimedon compressa*, *Amphimedon viridis*, *Aplysina fulva*, *Aplysina lactuca*, and *Mycale* sp.) inhibited all three bacterial strains, accounting for 21.7% of the active organic extracts and 38.5% of the sponge samples tested. Representative results from these assays are shown in Figure 4.

In total, seven sponge species exhibited antibacterial activity against all three bacterial strains tested, representing 9% of the organisms collected. Of these, four species were active in both aqueous and organic extractions, corresponding to 5% of the total samples.

## 4. Discussion

Bioprospecting marine organisms for antibacterial activity remains a critical strategy in the search for novel bioactive compounds with biotechnological and therapeutic potential. Extraction methods play a fundamental role in the recovery of such compounds, ranging from simple aqueous extractions to organic solvents with varying polarities [9,11,13,15]. In the present study, alongside distilled water, we employed 50% acetonitrile due to its compatibility with both polar and non-polar substances, as well as its documented effectiveness in reversed-phase HPLC for biomolecule recovery [16,17,18]. This choice was intended to support future fractionation and compound identification efforts.

Our findings revealed that 30.7% of the tested marine organisms exhibited antibacterial activity, particularly in organic extracts. While aqueous extracts from seaweeds were inactive, sponge extracts demonstrated activity across both solvent types. These results corroborate previous reports [13,15,19,20] and underscore the relevance of solvent polarity in recovering lipophilic secondary metabolites, many of which are linked to antimicrobial effects. For instance, although *Haliclona* species showed activity in studies using methanol or dichloromethane [21,22,23,24,25], our acetonitrile-based extraction did not yield active compounds from these sponges, highlighting the influence of solvent choice.

Geographical variation may also influence metabolite profiles. For example, *Aplysina fulva* and *Amphimedon viridis* were previously reported as inactive when extracted with acetone [26] but showed activity in our acetonitrile extracts. Similarly, the aqueous extract of *Ircinia felix* exhibited Gram-positive activity in our study. These differences may reflect the environmental or ecological factors that modulate secondary metabolite production [16,27].

Among all of the tested taxa, sponges from the genus *Aplysina* demonstrated the most consistent and potent antibacterial activity, in agreement with previous studies [28]. Other sponge genera, including *Agelas*, *Amphimedon*, and *Mycale*, also showed significant activity, particularly in organic extracts. This aligns with prior reports highlighting their alkaloid-rich profiles and broad-spectrum bioactivity [29,30,31,32,33]. In contrast, *Pseudosuberites* sp. displayed higher activity in aqueous extracts, reinforcing the importance of solvent compatibility and species-specific metabolite solubility [34].

Notably, extracts from *Topsentia ophiraphidites* and *Tedania ignis* were inactive against *E. coli*, consistent with some previous findings but contrasting others [21,34,35]. These differences further underscore the influence of extraction methods and solvents on antibacterial efficacy.

Regarding macroalgae, organic extracts were more effective, particularly against Gram-positive bacteria. *Amansia multifida* organic extract showed the most pronounced activity, corroborating earlier findings using hexane-based extractions [36]. Extracts from *Dictyota* spp. and *Sargassum* spp. also showed inhibitory effects, which have been attributed to sterol content and other lipophilic compounds [37,38]. In contrast, several green and red algae, including *Ulva fasciata*, *Caulerpa prolifera*, and *Gracilaria domingensis*, lacked antibacterial activity in our study, supporting prior reports [39].

Overall, our results reinforce the potential of marine organisms—particularly sponges—as valuable sources of antibacterial compounds. Several extracts demonstrated significant activity, especially those obtained with organic solvents, highlighting the influence of solvent polarity on the extraction of bioactive metabolites [32,33,34,35,40,41]. The genus *Aplysina* stood out as a particularly promising candidate, along with the species of *Amphimedon*, *Agelas*, and *Mycale*, all of which yielded broad-spectrum antibacterial activity.

A general trend observed across our assays was the greater susceptibility of Gram-positive bacteria to the tested extracts. This can likely be attributed to structural differences in the bacterial cell envelope: the simpler peptidoglycan-rich wall of Gram-positive bacteria may facilitate the entry of bioactive compounds, whereas the outer membrane of Gram-negative bacteria acts as a permeability barrier that limits compound access [42,43,44].

These findings are especially relevant in light of the escalating public health threat posed by antimicrobial resistance, which has been linked to millions of deaths globally each year [45]. The identification of new antibacterial sources from marine biodiversity—particularly from underexplored tropical regions—contributes meaningfully to the global effort to discover alternative therapeutic agents.

While this study was designed as an initial screening effort, it provides essential insights into the antibacterial potential of marine organisms and lays the groundwork for future investigations. The use of disk diffusion assays allowed for the rapid assessment of a large number of extracts, enabling the identification of promising candidates for a more detailed analysis. Building on these results, future studies will aim to isolate and chemically characterize the active compounds, assess their stability and potential cytotoxicity, and explore their mechanisms of action. Such efforts will be instrumental in advancing these findings toward practical applications in biotechnology and antimicrobial therapy.

## 5. Conclusions

This study highlights the biotechnological potential of marine organisms from the northeastern coast of Brazil as sources of antibacterial compounds. Approximately 30.7% of the tested extracts exhibited inhibitory activity, with organic extracts demonstrating greater efficiency. Sponges from the genera *Aplysina* and *Amphimedon*, as well as the species *Mycale* sp. and *Pseudosuberites* sp., were the most promising, while *Amansia multifida* was the most notable among marine algae, showing a significant inhibition halo against *Staphylococcus aureus*.

## Data Availability

No new data were created or analyzed in this study. Data sharing is not applicable to this article.

## Supplementary Materials

The following supporting information can be downloaded at: https://www.mdpi.com/article/10.3390/microorganisms13040940/s1, Table S1—Marine organisms collected from Parque da Pedra da Risca do Meio, Ceará. Table S2—Marine organisms collected from Pacheco Beach, Ceará. Table S3—Marine organisms collected from Paracuru Beach, Ceará. Table S4—Results of the disk diffusion antibiogram assay for aqueous extracts of marine organisms—average inhibition zone diameter (mm). Table S5—Results of the disk diffusion antibiogram assay for organic extracts of marine organisms—average inhibition zone diameter (mm).
