# Supplementary material for: Bioprospecting of Marine Organisms: Exploring Antibacterial Activities in Aqueous and Organic Extracts
Source: Microorganisms. 2025 Apr 18;13(4):940. doi: 10.3390/microorganisms13040940 (PMC12029734; doi:10.3390/microorganisms13040940)
Supplement: Supplementary file 1 [file microorganisms-13-00940-s001.zip › microorganisms-3557629-supplementary.pdf]

"BIOPROSPECTING OF MARINE ORGANISMS: EXPLORING ANTIBACTERIAL ACTIVITIES IN AQUEOUS AND ORGANIC EXTRACTS"

Vinícius Paulino Pinto Menezes[1]; Aldeni Moreira da Silva Filho[1]; Aline Jeferson Costa[1]; Ulisses Santos Pinheiro[2]; Renata Pinheiro Chaves[1]; Alexandre Lopes Andrade[3]; Mayron Alves de Vasconcelos[3,4]; Edson Holanda Teixeira[3]; Alexandre Holanda Sampaio[1]; Celso Shiniti Nagano[1]; Rômulo Farias Carneiro[1*].

1. Universidade Federal do Ceará, Departamento de Engenharia de Pesca, Laboratório de Biotecnologia Marinha - BioMar, Av. Humberto Monte, s/n, Campus do Pici, bloco 871, 60440-970 Fortaleza, CE, Brazil.
2. Universidade Federal de Pernambuco, Departamento de Zoologia, Av. Prof. Moraes Rego, 1235, Cidade Universitária, 50670-901 Recife, PE, Brazil.
3. Universidade Federal do Ceará, Departamento de Patologia e Medicina Legal, Laboratório Integrado de Biomoléculas - LIBS, Av. Monsenhor Furtado, s/n, 60430-160 Fortaleza, CE, Brazil.
4. Universidade Estadual do Ceará, Faculdade de Educação de Itapipoca, Av. da Universidade, s/n, 62500-000, Madalenas, Itapipoca, CE, Brazil.

[*] Correspondence to: Rômulo Farias Carneiro, e-mail: romulofc2603@gmail.com

Table S1 – Marine organisms collected from Parque da Pedra da Risca do Meio, Ceará.

Marine Organism	Phylum	Species
Marine Sponges	Porifera	*Aiolochroia crassa*
		Agelas sp.
		Agelas sventres
		Amphimedon compressa
		Aplysina cauliformis
		Aplysina fistularis
		Aplysina lactuca
		Callyspongia vaginalis
		Clathria nicoleae
		Erylus formosus
		Geodia sp.
		Ircinia strobilina
		Mycale sp.
		Niphates erecta
		Pseudosuberites sp.
		Topsentia ophiraphidites

Table S2 – Marine organisms collected from Pacheco Beach, Ceará.

Marine Organism	Phylum	Species
Marine Sponges	Porifera	*Amphimedon viridis*
		Aplysina fulva
		Haliclona implexiformis
		Ircinia felix
		Tethya sp.
Marine Macroalgae	Chlorophyta	*Caulerpa cupressoides*
		Caulerpa prolifera
		Caulerpa racemosa
		Caulerpa sertularioides
		Ulva fasciata
		Ulva lactuca
	Phaeophyta	*Dictyopteris delicatula*
		Lobophora variegata
	Rhodophyta	*Amansia multifida*
		Botryocladia occidentalis
		Cryptonemia crenulata
		Cryptonemia luxurians
		Cryptonemia sp.
		Dictyurus occidentalis
		Gracilaria domingensis
		Gracilariopsis sjoestedtii
		Halymenia sp.
		Hypnea musciformis
		Osmundaria obtusiloba
		Pterocladiella capillacea
		Solieria filiformis

Table S3 – Marine organisms collected from Paracuru Beach, Ceará.

Marine Organism	Phylum	Species
Marine Sponges	Porifera	*Chondrilla caribensis*
		Cinachyrella alloclada
		Haliclona caerulea
		Haliclona melana
		Placospongia sp.
		Tedania ignis
Marine Macroalgae	Chlorophyta	*Anadyomene stellata*
		Bryopsis pennata
		Bryopsis sp.
		Caulerpa mexicana
		Codium isthmocladum
		Dictyosphaeria cavernosa
		Enteromorpha prolifera
		Udotea flabellum
		Valonia aegagropila
	Phaeophyta	*Dictyota dichotoma*
		Dictyota mertensii
		Padina gymnospora
		Sargassum vulgare
		Spatoglossum schroederi
	Rhodophyta	*Acanthophora spicifera*
		Bryothamnion seaforthii
		Bryothamnion triquetum
		Corallina panizzoi
		Corynomorpha clavata
		Digenea simplex
		Galaxaura sp.
		Gelidiella acerosa
		Gracilaria cervicornis
		Gracilaria ferox
		Gracilaria ramosíssima
		Gracilaria wrightii
		Gracilaria sp.
		Laurencia sp.
		Meristiella echinocarpum
		Ochtodes seundiramea

Table S4 – Results of the disk diffusion antibiogram assay for aqueous extracts of marine organisms – average inhibition zone diameter (mm).

Marine Organism	Species	Bacterial strains		
		E. coli	***S. aureus***	***S. epidermidis***
Marine Sponges	*Agelas sventres*	0.00±0.00 A	9.00±1.00 D	0.00±0.00 A
	Amphimedom compressa	8.67±0.58 B	9.67±0.58 D	8.67±1.15 B
	Amphimedon viridis	8.00±0.00 B	0.00±0.00 A	8.00±0.00 B
	Aplysina fistularis	10.00±0.00 C	14.00±0.00 C	13.00±1.00 C
	Aplysina fulva	11.33±1.15 D	15.00±1.73 C	14.00±2.00 C
	Aplysina lactuca	12.33±1.53 D	14.00±4.00 C	15.33±2.31 C
	Mycale sp.	7.00±0.00 B	11.33±2.52 E	8.67±1.15 B
	Pseudosuberites sp.	15.00±1.00 F	19.33±2.31 F	14.67±2.31 G
	Tedania ignis	0.00±0.00 A	15.67±1.53 C	9.33±1.15 D
	Topsentia ophiraphidites	0.00±0.00 A	16.00±0.00 C	10.67±1.15 D
Positive Control	Ampicillin	21.00±1.00 E	22.00±1.00 B	24.00±0.00 E
Negative Control	Distilled water	0.00±0.00 A	0.00±0.00 A	0.00±0.00 A

Diameter of inhibition zone including disk diameter of 6 mm at a volume of 10 µL of extract, water (negative control), or ampicillin (50 µg; positive control) /disk. Means with different letters within a column are significantly different ($p < 0.05$).

Table S5 – Results of the disk diffusion antibiogram assay for organic extracts of marine organisms – average inhibition zone diameter (mm).

**Marine Organism**	**Species**	**Bacterial strains**		
		E. coli	***S. aureus***	***S. epidermidis***
Marine Sponges	*Agelas sventres*	0.00±0.00 A	15.00±1.00 C	12.33±0.58 H
	Aiolochroia crassa	0.00±0.00 A	9.33±1.15 B	0.00±0.00 A
	Amphimedon compressa	9.00±1.00 B	10.67±0.58 B	9.33±1.15 B
	Amphimedon viridis	9.00±1.00 B	9.00±1.00 B	9.33±0.58 B
	Aplysina cauliformes	15.67±2.89 C	16.33±5.13 C	0.00±0.00 A
	Aplysina fistularis	14.33±0.58 C	16.00±2.00 C	0.00±0.00 A
	Aplysina fulva	16.33±1.15 C	23.00±2.00 D	18.00±0.00 E
	Aplysina lactuca	16.33±1.53 C	19.00±4.58 F	8.33±0.58 B
	Erylus formosus	0.00±0.00 A	0.00±0.00 A	15.00±1.00 F
	Ircinia felix	0.00±0.00 A	10.33±0.58 B	8.00±1.00 B
	Mycale sp.	16.33±1.15 C	22.67±4.16 D	18.33±0.58 D
	Pseudosuberites sp.	17.67±0.58 E	0.00±0.00 A	0.00±0.00 A
	Topsentia ophiraphidites	0.00±0.00 A	18.33±0.58 G	14.00±0.00 G
Marine Macroalgae	*Amansia multifida*	0.00±0.00 A	24.00±4.00 E	0.00±0.00 A
	Corallina panizzoi	0.00±0.00 A	0.00±0.00 A	9.00±0.00 B
	Cryptonemia crenulata	0.00±0.00 A	0.00±0.00 A	10.00±0.00 B
	Dictyota dichotoma	0.00±0.00 A	8.00±0.00 B	0.00±0.00 A
	Dictyota mertensii	0.00±0.00 A	8.00±0.00 B	8.33±0.58 B
	Gracilaria ramosissima	0.00±0.00 A	0.00±0.00 A	8.67±0.58 B
	Gracilaria sp.	0.00±0.00 A	9.33±0.58 B	8.67±0.58 B
	Laurencia sp.	0.00±0.00 A	0.00±0.00 A	9.00±0.00 B
	Sargassum vulgare	0.00±0.00 A	11.00±2.00 H	0.00±0.00 A
	Valonia aegagropila	0.00±0.00 A	12.00±1.00 I	0.00±0.00 A
Positive Control	Ampicillin	20.00±0.00 D	23.00±0.00 D	22.00±3.00 C
Negative Control	Evaporated acetonitrile	0.00±0.00 A	0.00±0.00 A	0.00±0.00 A

Diameter of inhibition zone including disk diameter of 6 mm at a volume of 10 µL of extract, water (negative control), or ampicillin (50 µg; positive control) /disk. Means with different letters within a column are significantly different ($p < 0.05$).
